# Openness and age influence cognitive progression: a longitudinal study

**DOI:** 10.1055/s-0043-1775884

**Published:** 2023-10-29

**Authors:** Silvia Stahl Merlin, Sonia Maria Dozzi Brucki

**Affiliations:** 1 Universidade de São Paulo, Departamento de Neurologia, Unidade de Neurologia Cognitiva e Comportamental, São Paulo SP, Brazil.

**Keywords:** Cognitive Dysfunction, Dementia, Personality, Lifestyle, Disfunção Cognitiva, Demência, Personalidade, Estilo De Vida

## Abstract

**Background** Some psychological and personality characteristics of individuals seem to determine behavioral patterns that are associated with better health throughout life and, consequently, prevent the progression of early cognitive changes to dementia.

**Objective** To identify which individuals have modified cognitive ratings after 24 months of follow-up and correlating with personality traits.

**Methods** One hundred and two volunteers were evaluated clinically and for personality characteristics and neuropsychological testing. Of these, 25 subjects were classified as cognitively normal (CN), 25 as subjective cognitive decline (SCD), 28 as nonamnestic mild cognitive impairment (naMCI), and 24 as amnestic mild cognitive impairment (amMCI) at baseline. Follow-up occurred over 2 years from the initial assessment, and the cognitive categories of the participants were re-analyzed every 6 months to observe differences in their classification.

**Results** Out of the 102 subjects, 65 remained at follow-up. The sample followed-up longitudinally was composed predominantly of women (65%), white (74%), with a mean age of 78 (±7.5) years old and 12 (±4.8) years of schooling. Throughout the process, 23% of CN, 15% of SDC, and 27% of naMCI individuals worsened cognitively. Amnestic with mild cognitive impairment volunteers remained stable or improved. Individuals with older age show more significant cognitive deterioration, and those with very low or high rates of the openness personality trait are associated with cognitive decline utilizing the Fisher exact test, probably because the open extremes influence choices, stress management, and behavioral maintenance.

**Conclusion** The factors most associated with cognitive change in this group of older adults were age and the intensity of the openness aspects of personality.

## Resumo

**Antecedentes** Algumas características psicológicas e de personalidade determinam padrões comportamentais que se associam a uma melhor saúde ao longo da vida e, consequentemente, impedem a progressão de alterações cognitivas para demência.

**Objetivo** Identificar quais indivíduos modificaram cognitivamente após 24 meses de acompanhamento e correlacionar com traços de personalidade.

**Métodos** 102 voluntários foram avaliados clinicamente por características de personalidade e testes neuropsicológicos. Destes, 25 indivíduos foram classificados como cognitivamente normais (CN), 25 como com declínio cognitivo subjetivo (DCS), 28 com comprometimento cognitivo leve não amnéstico (CCLNa) e 24 com comprometimento cognitivo leve amnéstico (CCLAm) no início do estudo. O acompanhamento ocorreu ao longo de 2 anos a partir da avaliação inicial, e as categorias cognitivas dos participantes foram reanalisadas a cada 6 meses para observar diferenças em sua classificação.

**Resultados** Dos 102 indivíduos, 65 permaneceram em acompanhamento. A amostra acompanhada longitudinalmente foi composta predominantemente por mulheres (65%), brancas (74%), com média de idade de 78 (±7,5) anos e 12 (±4,8) anos de escolaridade. Ao longo do processo, 23% dos indivíduos CN, 15% dos DCS e 27% dos indivíduos CCLNa pioraram cognitivamente. Os voluntários CCLAm permaneceram estáveis ou melhoraram. Indivíduos com idade mais avançada apresentam deterioração cognitiva mais significativa, e aqueles com taxas muito baixas ou altas do traço de personalidade abertura estão associados ao declínio cognitivo utilizando o teste exato de Fisher. Provavelmente, a característica abertura influencia as escolhas, o gerenciamento do estresse e a manutenção do comportamento.

**Conclusão** Os fatores mais associados à alteração cognitiva neste grupo de idosos foram a idade e a intensidade dos aspectos abertura da personalidade.

## INTRODUCTION

Population aging justifies studies on chronic degenerative conditions such as neuropsychiatric disorders, as these are frequent in the elderly population. 1 The understanding of anticipatory conditions to dementia, especially mild cognitive impairment (MCI), is growing in medical practice with a preventive diagnostic function of neurodegenerative conditions. 2

To date, studies on the characterization of risk factors for dementia and protective factors for the maintenance of healthy aging have been more frequent in determining cognitive evolution. 3 Thus, the association between the worse cognitive outcome in individuals with hypertension, diabetes, obesity, smoking, physical inactivity, and neuropsychiatric symptoms has already been evidenced. 4 Similarly, certain individual psychological and personality characteristics determine behavioral patterns and are associated with better health throughout life, preventing the progression of prodromal states to dementia. 5 6

Mild cognitive impairment is defined as a cognitive complaint confirmed by an objective assessment of the different cognitive domains with preserved functionality. 7 8 The annual risk of MCI progression to dementia is estimated at 5 to 15% compared with a rate of 1% in cognitively normal elderly individuals. 9 Its rates are lower in younger individuals and increase with age. However, some individuals with MCI never progress to dementia syndromes. 10

Although neurodegeneration may be the most frequent cause of MCI, cognitive deterioration may evolve due to other clinical conditions, such as cardiovascular or infectious diseases, as well as other factors, such as educational levels and cultural or personality traits. 11

Another similar condition is subjective cognitive decline (SCD), which refers to an individual's awareness of cognitive decline, compared with previous cognitive status without a trigger and without change in cognitive tests, as occurs in MCI. 12

Some studies indicate that such subjective memory complaints are more strongly related to depression, sleep disorders, adverse effects of medication or personality traits. 13 It is believed that personality traits have biological bases, reflecting neurophysiological processes mediated by brain networks. 14 Personality traits are also known to be highly inheritable, even though personality also results from interactions between the brain and the environment. 15 The most widespread explanatory model of personality used in research, known as the *Big Five* , refers to the personality structure as the individual fusion of five major factors: neuroticism (susceptibility to stress), agreeableness (interpersonal interaction and empathy), conscientiousness (control and motivation to achieve goals), extroversion (need for stimulation, the existence of self-confidence and spontaneity), and openness (exploratory behaviors to experiences and reflective behavior). 17

Research indicates a relationship between MCI and personality traits such as neuroticism, openness, and conscientiousness. There are also data showing that neuroticism scores increase and openness scores decrease in MCI cases that progress to dementia. 18

Understanding the relationship between personality and cognitive ability in older adults is considered important since personality traits affect cognitive decline mainly by interfering with cognitive reserve, which is an active process that is closely related to motivation, interest, and intensity of effort, characteristics that result from personality traits. 19 20 Our aim is to observe, after 24 months of follow-up, which factors interfere with the evolution of cognitive disorders in the MCI patients.

## METHODS

**Figure 1:**
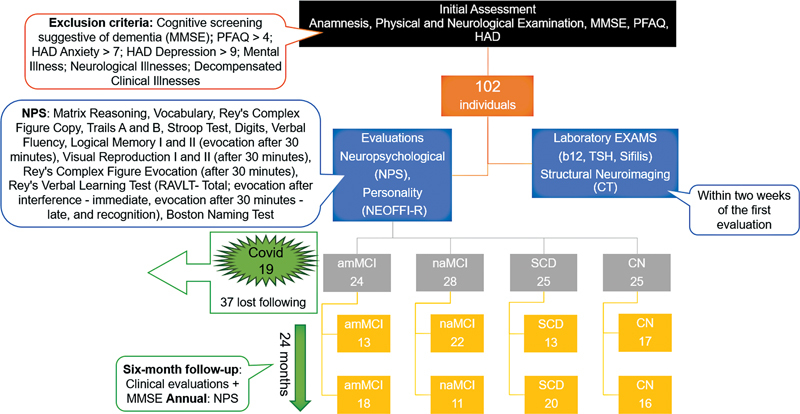
Flowchart of the study population. Abbreviations: amMCI, amnestic mild cognitive impairment; CN, cognitively normal; HAD, Hospital Anxiety and Depression Screening Questionnaire; MMSE, Mini-Mental State Examination; naMCI, non-amnestic mild cognitive impairment; NEO-FFI-R, Personality Inventory NEO-Revised; PFAQ, Pfeffer Functional Activities; SCD, subjective cognitive decline.

This is a longitudinal study with volunteers > 60 years old who participate in the Brazilian Aging Memory Study (BRAMS), of the Hospital das Clínicas of the Faculdade de Medicina of the Universidade de São Paulo (HC/FMUSP, in the Portuguese acronym). The study included 102 individuals from January 2017 to January 2019, who were classified and evaluated according to the flowchart described in Figure 1 after signing the Free and Informed Consent form. The data were collected prospectively for 24 months, and at the end of the segment, 65 older adults remained in the follow-up.

We excluded from the study individuals with a) functional changes, assessed by the Pfeffer Functional Activities Questionnaire (PFAQ > 4); b) cognitive screening suggestive of dementia, tested with scores from the Mini-Mental State Examination (MMSE) and the Brief Cognitive Screening Battery; c) predominant symptoms of anxiety and depression, as assessed by the Hospital Anxiety and Depression Scale (HAD anxiety > 7; HAD depression > 9); d) a psychiatric illness; e) a decompensated clinical illness; and f) antecedents or signs of neurological diseases.

The patients underwent at least two comprehensive neuropsychological assessments and the criteria used for categorizing the sample were Petersen/Winblad, 7 and/or Jak/Bondi. 21 For both criteria, participants with MCI were also classified as “amnestic” when memory was impaired, or as “nonamnestic” when one or more domains except memory were impaired. Individuals classified as SCD were those with cognitive complaints, but with neuropsychological test results within normal parameters for age and education. Cognitively normal (CN) elderlies were those without cognitive complaints or changes in cognitive tests. After each evaluation, the participants were reclassified as to their cognitive diagnosis, which could remain the same, be progressive for cognitive worsening, or reversible (with cognitive improvement).

The personality assessment was performed using the NEO Personality Inventory-Revised (NEO-FFI-R), based on the Big Five theory, which uses the models of extroversion, neuroticism, amiability, conscientiousness, and openness as the main characteristics ( **Supplementary Material** - https://www.arquivosdeneuropsiquiatria.org/wp-content/uploads/2023/07/ANP-2023.0001-Supplementary-Material.pdf). ^22^

The computer system of the test publisher performed the scoring of the NEO-FFI-R. The computer score system transforms the numerical rating of the test into qualitative categories ranging from very low to very high. Values are available in Table 1 . Structural neuroimaging scans were acquired by tomography. 23

**Table 1:** Inference of variables for negative evolution after 24 months

Variable		Cognitive weakness - Negative conversion		*p-value*
		No	Yes	
Age (years old) - Mean (CI)		77.6 (75.77–79.44)	81.91 (75.14–88.68)	0.05*
Schooling (years) - Mean (CI)		12.42 (11.14–13.69)	14.55 (10.94–18.16)	0.27
MMSE (points) - Mean (CI)		28.4 (27.92–28.87)	28.18 (27.19–29.17)	0.42
IQ (points) - Mean (CI)		104.13 (100.64–107.62)	101.45 (93.91–109)	0.61
HADD - Mean (CI)		3.52 (2.7–4.34)	4.8 (2.42–7.18)	0.25
HADANS - Mean (CI)		3.84 (3.02–4.66)	4.1 (2.24–5.96)	0.62
Sex ( *n* = 65)	Male ( *n* = 23)	70% (16)	30% (7)	0.09
	Female ( *n* = 42)	88% (37)	12% (5)	
Age ( *n* = 65)	Caucasian ( *n* = 49)	79% (39)	21% (10)	0.85
	Black ( *n* = 7)	86% (6)	4% (1)	
	Eastern ( *n* = 9)	88% (8)	12% (1)	
Hypertension ( *n* = 65)	Present ( *n* = 34)	23% (8)	77% (26)	0.35
	Absent ( *n* = 31)	87% (27)	13% (4)	
Diabetes ( *n* = 65)	Present ( *n* = 13)	85% (11)	15% (2)	1.0
	Absent ( *n* = 52)	81% (42)	19% (10)	
Dyslipidemia ( *n* = 65)	Present ( *n* = 26)	77% (20)	23% (6)	0.52
	Absent ( *n* = 39)	84% (33)	16% (6)	
Hypothyroidism ( *n* = 64)	Present ( *n* = 11)	100% (11)	0	0.1
	Absent ( *n* = 53)	77% (41)	23% (12)	
Cardiopathy ( *n* = 65)	Present ( *n* = 17)	70% (12)	30% (5)	0.16
	Absent ( *n* = 48)	85% (41)	15% (7)	
Neoplasia ( *n* = 65)	Present ( *n* = 5)	80% (4)	20% (1)	1.0
	Absent ( *n* = 60)	81%(49)	19% (11)	
Opening ( *n* = 65)	Very low ( *n* = 11)	64% (7)	36% (4)	0.0005*
	Low ( *n* = 22)	95% (21)	5% (1)	
	Mean ( *n* = 24)	92% (22)	8% (2)	
	High ( *n* = 8)	37% (3)	63% (5)	
	Very high ( *n* = 0)	0% (0)	0% (0)	
Amability ( *n* = 65)	Very low ( *n* = 0)	0% (0)	0% (0)	0.4
	Low ( *n* = 13)	85% (11)	15% (2)	
	Mean ( *n* = 35)	77% (27)	23% (8)	
	High ( *n* = 14)	93% (13)	7% (1)	
	Very high ( *n* = 3)	67% (2)	33% (1)	
Consciousness ( *n* = 65)	Very low ( *n* = 2)	100% (2)	0	0.35
	Low ( *n* = 5)	60% (3)	40% (2)	
	Mean ( *n* = 36)	83% (30)	17% (6)	
	High ( *n* = 20)	85% (17)	15% (3)	
	Very high ( *n* = 2)	50% (1)	50% (1)	
Extroversion ( *n* = 65)	Very low ( *n* = 5)	80% (4)	20% (1)	0.9
	Low ( *n* = 21)	76% (16)	24% (5)	
	Mean ( *n* = 25)	84% (21)	15% (4)	
	High ( *n* = 14)	86% (12)	14% (2)	
	Very high ( *n* = 0)	0% (0)	0% (0)	
Neuroticism ( *n* = 65)	Very low ( *n* = 17)	77% (13)	23% (4)	0.79
	Low ( *n* = 27)	85% (23)	15% (4)	
	Mean ( *n* = 18)	78% (14)	22% (4)	
	High ( *n* = 3)	100% (3)	0% (0)	
	Very high ( *n* = 0)	0% (0)	0% (0)	
Cognitive group ( *n* = 65)	CN ( *n* = 17)	76% (13)	24% (4)	0.24
	SDC ( *n* = 13)	86% (11)	15% (2)	
	naMCI ( *n* = 22)	73% (16)	27% (6)	
	amMCI ( *n* = 13)	100% (13)	0% (0)	

Abbreviations: CI, confidence interval; HADANS, Hospital Depression and Anxiety Screening Questionnaire Anxiety Scope; HADD, Hospital Depression and Anxiety Screening Questionnaire Depression Scope; IQ, intelligence quotient; MMSE, Mini-Mental State Examination.

Note: *Statistically significant values.

For the statistical study, the correlation analyses were performed using the Spearman correlation coefficient and the verification of the null hypothesis, using the cutoff point value of *p* < 0.05 (5%). To detect significant differences in the variables between the different cognitive groups, analysis of variance (ANOVA) was performed for variables with normal distribution, and the Kruskal Wallis test for those not normally distributed. In the longitudinal evaluation, the cognitive improvement and worsening were calculated with t-tests (parametric) associated with normal variables and the Wilcoxon test for non-normal variables. Applying the Fisher exact test, tables of contingencies were used to verify the association of categorical variables. All participants signed the informed consent, and the present study was approved by the Ethical Committee (process no. 13640/2015).

## RESULTS

The participants had a mean age of 77.61 (7.6) years old, ranging from 62 to 96 years old, and 12.8 (4.8) years of schooling, being mostly women (62%) of white ethnicity (75%). After the neuropsychological assessment, the participants were classified as cognitively normal ( *n* = 25; 24.5%), SCD ( *n* = 25; 24.5%), naMCI (n = 28; 27.5%) and amMCI ( *n* = 24; 23.5%). Regarding personality characterization, we observed no differences between the cognitive groups.

**Figure 2:**
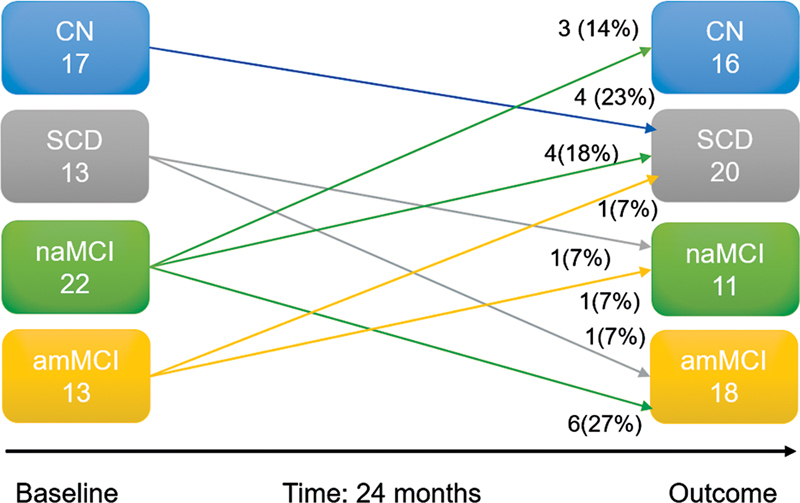
Demonstration of heterogeneity of the cognitive trajectories of elderly individuals followed-up for 24 months.

Over the 2-year follow-up, shown in Figure 2 , it was possible to observe the changes in classification between the groups, and the heterogeneity of the cognitive trajectory of these elderly subjects. The classification remained stable in 67.5% of the subjects, while 18.5% showed cognitive worsening, and 14% showed cognitive improvement.

The contingency table showed the important association of two factors with cognitive worsening: age and the characteristic openness detected by the personality inventory. For age, the higher the value, the greater the expressiveness of the conversion. Regarding openness, it was observed that very low openness and high openness reduced cognitive worsening. The inferences of the variables related to cognitive worsening are shown in Table 1 .

## DISCUSSION

Our findings revealed that very low openness and high openness reduced cognitive worsening, and age was associated with a change in diagnoses. They are consistent with the current literature, which describes advanced age as an established risk of worsening cognitive performance due to greater brain vulnerability. 24 It is known that the prevalence rate of dementia doubles every 6 years, starting at 65, affecting 40% of people > 90 years old. In addition to structural brain issues, depletion of cognitive reserves and progressive social isolation, common in the elderly, also occur. 25

Regarding personality traits, studies have shown that individuals with MCI who progressed to dementia have increased neuroticism scores and decreased openness scores, according to data computed by the NEO-PI-R personality instrument. 26 27 28 More specifically, in a 2-year follow-up study, it was observed that among 510 healthy individuals aged ∼ 51 years old, neuroticism scores increased, and openness scores decreased in MCI cases that progressed to dementia. 18

Another relationship was elucidated with a cross-sectional study, with 44 cognitively normal individuals, 57 patients with MCI, and 9 with AD, when the openness characteristic correlated positively with the MMSE score. That is, the higher the score in the behavioral characteristic of interest in knowledge, curiosity, and search for novelty (characteristics of the openness aspect of personality), the higher the score in the cognitive evaluation. This fact can be understood considering that personality traits contribute to choices and lifestyle, influencing cognitive, social, and physical activities. 29

Openness is the personality trait that reflects creativity, mental flexibility, and abstract reasoning capacity, properties driven by the increased function of mesocortical networks. 30 Recent evidence has observed that tasks involving imagination, creativity, mental rambling, and fluid intelligence presuppose default mode network *(* DMN *)* connectivity activities. 31 These characteristics are inherent to the openness personality trait, which many studies evidence is related to the proportion of robustness, synchronized activity, and brain rest in the posterior cingulate and prefrontal regions. 32 It is known from several functional neuroimaging studies that the inactivation of frontal regions related to the DMN network was observed in participants with MCI compared with controls and even more significantly in AD patients. 33

Another perspective proposes that higher values of openness do not necessarily lead to significant benefits. 34 Although there is no robust scientific evidence, it can be suggested that excessive openness refers to less persistent subjects and those whose lifestyle is constantly modified, causing scarcity of routines and behavioral patterns. Excessing this could hinder the establishment of protective attitudes to cognitive health, including physical activity practices and sleep and eating routines. 29 30 35 People with higher scores on openness are more likely to engage in irresponsible or thoughtless behavior. They can also go against established social norms and have a need for immediate satisfaction, regardless of the consequences.

Thus, low, and high openness would make it difficult to maintain social contacts, establish new cognitive and physical challenges, and overcome the resistance that emerges throughout life. 36

As for other personality traits, unlike previous literature, the present sample did not show neuroticism as a factor of significant association with the cognitive evolution of individuals. This could be explained by the fact that the older adults in the sample were initially selected after the HAD screening, which addresses depressive and anxiety symptoms (contents addressed by the neuroticism dimension).

Meanwhile, it is clear that lifestyle factors determined by personality traits can modify the incidence of cognitive disorders. 22 This is because personality influences motivation, interest, and effort intensity that determines the stress response, health behavior choices, and cognitive stimulation activity of individuals with MCI. 14

That is, personality is associated with dementia risk when factors that mediate this association are considered, such as personality influences choices, stress management, and behavioral maintenance. 30

The classification changes between the spectrum, including normal cognition at one end and dementia at the other, may occur because heterogeneous etiologies cause MCI with different forms of appreciation. Thus, until the degenerative mechanisms stand out, fluctuations between the spectrum are expected. 31

Although the results of the present study show an association between the age and openness variables for worse cognitive evolution, interpreting the predictors of cognitive evolution in the elderly is still developing. In an attempt to associate which factors are linked to prognosis, we perhaps mistakenly try to simplify highly heterogeneous situations. 37

Despite the above contributions, the present study has certain limitations. First, the study sample primarily comprises women (62%). This is common in samples of Brazilian older adults, possibly because women are more concerned about health, more easily accept going to the health service, and thus have a higher life expectancy. 38 Second, the majority of the sample is white (75%), a class that had more social and educational opportunities in the past, justifying the high level of schooling found (∼ 13 years) compared with the Brazilian population, which has 9.06 years of schooling for the general population. 39

Finally, the loss of individuals in the research occurred for several reasons. Still, it was mainly due to the COVID-19 pandemic, which limited the elderly to perform re-evaluations and longitudinal follow-ups. Despite these limitations, we showed the first study of personality traits and MCI in Brazil, with a long-term follow-up with consecutive clinical and neuropsychological evaluations.

In conclusion, the 24-month follow-up of elderly individuals with different cognitive ratings was able to demonstrate that the most important factors for grouping changes were age (the older the individual, the lower the chance of cognitive improvement), and personality characteristics related to openness (search for novelty, creativity, and flexibility) in moderate proportions.

Despite the longitudinal design, complications led to the reduction of the final sample, limiting the robustness of statistical findings. For this reason, it is suggested to expand the number of participants and follow-up time, aiming at a better understanding of the interference of psychological and behavioral factors in the protection and evolution of mild cognitive disorders.

## References

[1] Díaz-Mardomingo M DC García-Herranz S Rodríguez-Fernández R Venero C Peraita H Problems in Classifying Mild Cognitive Impairment (MCI): One or Multiple Syndromes? Brain Sci 2017 7 09 111 10.3390/brainsci7090111 28862676 PMC5615252

[2] von Gunten A Pocnet C Rossier J The impact of personality characteristics on the clinical expression in neurodegenerative disorders–a review Brain Res Bull 2009 80(4-5):179 191 10.1016/j.brainresbull.2009.07.004 19616079

[3] Knopman D S Beiser A Machulda M M Spectrum of cognition short of dementia: Framingham Heart Study and Mayo Clinic Study of Aging Neurology 2015 85 19 1712 1721 10.1212/WNL.0000000000002100 26453643 PMC4653114

[4] Alzheimer's Disease Neuroimaging Initiative David N D Lin F Porsteinsson A P Trajectories of Neuropsychiatric Symptoms and Cognitive Decline in Mild Cognitive Impairment Am J Geriatr Psychiatry 2016 24 01 70 80 10.1016/j.jagp.2015.06.001 26525995 PMC4691566

[5] Wilson R S Krueger K R Gu L Bienias J L Mendes de Leon C F Evans D A Neuroticism, extraversion, and mortality in a defined population of older persons Psychosom Med 2005 67 06 841 845 10.1097/01.psy.0000190615.20656.83 16314587

[6] Wilson R S Schneider J A Arnold S E Bienias J L Bennett D A Conscientiousness and the incidence of Alzheimer disease and mild cognitive impairment Arch Gen Psychiatry 2007 64 10 1204 1212 10.1001/archpsyc.64.10.1204 17909133

[7] Petersen R C Doody R Kurz A Current concepts in mild cognitive impairment Arch Neurol 2001 58 12 1985 1992 10.1001/archneur.58.12.1985 11735772

[8] Petersen R C Mild cognitive impairment as a diagnostic entity J Intern Med 2004 256 03 183 194 10.1111/j.1365-2796.2004.01388.x 15324362

[9] Roberts R O Knopman D S Mielke M M Higher risk of progression to dementia in mild cognitive impairment cases who revert to normal Neurology 2014 82 04 317 325 10.1212/WNL.0000000000000055 24353333 PMC3929198

[10] Panza F D'Introno A Colacicco A M Current epidemiology of mild cognitive impairment and other predementia syndromes Am J Geriatr Psychiatry 2005 13 08 633 644 10.1176/appi.ajgp.13.8.633 16085779

[11] MCI Working Group of the European Consortium on Alzheimer's Disease (EADC) Portet F Ousset P J Visser P J Mild cognitive impairment (MCI) in medical practice: a critical review of the concept and new diagnostic procedure. Report of the MCI Working Group of the European Consortium on Alzheimer's Disease J Neurol Neurosurg Psychiatry 2006 77 06 714 718 10.1136/jnnp.2005.085332 16549412 PMC2077456

[12] Jessen F Amariglio R E Buckley R F The characterisation of subjective cognitive decline Lancet Neurol 2020 19 03 271 278 10.1016/S1474-4422(19)30368-0 31958406 PMC7062546

[13] Van Harten A C Mielke M M Swenson-Dravis D M Subjective cognitive decline and risk of MCI Neurology 2018 91 e300 e312 29959257 10.1212/WNL.0000000000005863 PMC6070384

[14] Dubois J Galdi P Han Y Paul L K Adolphs R Resting-state functional brain connectivity best predicts the personality dimension of openness to experience Personal Neurosci 2018 1 e6 10.1017/pen.2018.8 30225394 PMC6138449

[15] KBASE Research Group Byun M S Jung J H Sohn B K Neuroticism, conscientiousness, and in vivo Alzheimer pathologies measured by amyloid PET and MRI Psychiatry Clin Neurosci 2020 74 05 303 310 10.1111/pcn.12983 31985106

[16] McAdams D P The five-factor model in personality: a critical appraisal J Pers 1992 60 02 329 361 10.1111/j.1467-6494.1992.tb00976.x 1635046

[17] Allik J The Almost Unbearable Lightness of Personality J Pers 2018 86 01 109 123 10.1111/jopy.12329 28545162

[18] Terracciano A Stephan Y Luchetti M Sutin A R Cognitive Impairment, Dementia, and Personality Stability Among Older Adults Assessment 2018 25 03 336 347 10.1177/1073191117691844 29214858 PMC5725278

[19] Curtis R G Windsor T D Soubelet A The relationship between Big-5 personality traits and cognitive ability in older adults - a review Neuropsychol Dev Cogn B Aging Neuropsychol Cogn 2015 22 01 42 71 10.1080/13825585.2014.888392 24580119

[20] Torrente F Pose M Gleichgerrcht E Personality changes in dementia: are they disease specific and universal? Alzheimer Dis Assoc Disord 2014 28 03 261 268 10.1097/WAD.0000000000000030 24614269

[21] Bondi M W Edmonds E C Jak A J Neuropsychological criteria for mild cognitive impairment improves diagnostic precision, biomarker associations, and progression rates J Alzheimers Dis 2014 42 01 275 289 10.3233/JAD-140276 24844687 PMC4133291

[22] Hauck Filho N Machado Wde L., Teixeira, M. A. P., Bandeira, D. R. Evidências de validade de marcadores reduzidos para a avaliação da personalidade no modelo dos cinco grandes fatores. Psicologia: Teoria e Pesquisa [online]. 2012, v. 28, n. 4 [Acessado 17 Julho 2021], pp. 417–423. Disponível em: <https://doi.org/10.1590/S0102-37722012000400007>. Epub 10 Jan 2013. ISSN 1806–3446.*https://doi.org/10.1590/S0102-37722012000400007*

[23] Coutinho A M Porto F H Duran F L Brain metabolism and cerebrospinal fluid biomarkers profile of non-amnestic mild cognitive impairment in comparison to amnestic mild cognitive impairment and normal older subjects Alzheimers Res Ther 2015 7 01 58 10.1186/s13195-015-0143-0 26373380 PMC4572657

[24] Livingston G Huntley J Sommerlad A Dementia prevention, intervention, and care: 2020 report of the Lancet Commission Lancet 2020 396(10248):413 446 10.1016/S0140-6736(20)30367-6 32738937 PMC7392084

[25] Jungwirth S Zehetmayer S Hinterberger M Tragl K H Fischer P The validity of amnestic MCI and non-amnestic MCI at age 75 in the prediction of Alzheimer's dementia and vascular dementia Int Psychogeriatr 2012 24 06 959 966 10.1017/S1041610211002870 22300486

[26] Hill N L Kolanowski A M Fick D Chinchilli V M Jablonski R A Personality as a moderator of cognitive stimulation in older adults at high risk for cognitive decline Res Gerontol Nurs 2014 7 04 159 170 10.3928/19404921-20140311-01 24635006 PMC4115044

[27] Ellendt S Voβ B Kohn N Predicting Stability of Mild Cognitive Impairment (MCI): Findings of a Community Based Sample Curr Alzheimer Res 2017 14 06 608 619 10.2174/1567205014666161213120807 27978792

[28] Nishita Y Tange C Tomida M Otsuka R Ando F Shimokata H Personality and global cognitive decline in Japanese community-dwelling elderly people: A 10-year longitudinal study J Psychosom Res 2016 91 20 25 10.1016/j.jpsychores.2016.10.004 27894458

[29] Caselli R J Dueck A C Locke D E Impact of Personality on Cognitive Aging: A Prospective Cohort Study J Int Neuropsychol Soc 2016 22 07 765 776 10.1017/S1355617716000527 27346168

[30] Toschi N Passamonti L Intra-cortical myelin mediates personality differences J Pers 2019 87 04 889 902 10.1111/jopy.12442 30317636 PMC6767500

[31] Boot N Baas M van Gaal S Cools R De Dreu C KW Creative cognition and dopaminergic modulation of fronto-striatal networks: Integrative review and research agenda Neurosci Biobehav Rev 2017 78 13 23 10.1016/j.neubiorev.2017.04.007 28419830

[32] Käckenmester W Bott A Wacker J Openness to experience predicts dopamine effects on divergent thinking Personal Neurosci 2019 2 e3 10.1017/pen.2019.3 32435738 PMC7219677

[33] Ouchi Y Kikuchi M A review of the default mode network in aging and dementia based on molecular imaging Rev Neurosci 2012 23 03 263 268 10.1515/revneuro-2012-0029 22752783

[34] Lesuis S L Hoeijmakers L Korosi A Vulnerability and resilience to Alzheimer's disease: early life conditions modulate neuropathology and determine cognitive reserve Alzheimers Res Ther 2018 10 01 95 10.1186/s13195-018-0422-7 30227888 PMC6145191

[35] Davis M Johnson S Estimating Alzheimer's Disease Progression Rates from Normal Cognition Through Mild Cognitive Impairment and Stages of Dementia Curr Alzheimer Res 2018 15 08 777 788 10.2174/1567205015666180119092427 29357799 PMC6156780

[36] Mazzeo S Padiglioni S Bagnoli S The dual role of cognitive reserve in subjective cognitive decline and mild cognitive impairment: a 7-year follow-up study J Neurol 2019 266 02 487 497 10.1007/s00415-018-9164-5 30604054

[37] Kivipelto M Solomon A Ahtiluoto S The Finnish Geriatric Intervention Study to Prevent Cognitive Impairment and Disability (FINGER): study design and progress Alzheimers Dement 2013 9 06 657 665 10.1016/j.jalz.2012.09.012 23332672

[38] César K G Brucki S M Takada L T Prevalence of Cognitive Impairment Without Dementia and Dementia in Tremembé, Brazil Alzheimer Dis Assoc Disord 2016 30 03 264 271 10.1097/WAD.0000000000000122 26629676

[39] Chaves M L Camozzato A L Godinho C Piazenski I Kaye J Incidence of mild cognitive impairment and Alzheimer disease in Southern Brazil J Geriatr Psychiatry Neurol 2009 22 03 181 187 10.1177/0891988709332942 19307320

